# Molecular Stratification of Antiphospholipid Syndrome Through Integrative Analysis of the Whole‐Blood RNA Transcriptome

**DOI:** 10.1002/art.70021

**Published:** 2026-02-13

**Authors:** Amala Ambati, Feiyang Ma, Katarina Kmetova, Sherwin Navaz, Claire K. Hoy, Cyrus Sarosh, Ajay Tambralli, Erika Navarro‐Mendoza, Johann E. Gudjonsson, J. Michelle Kahlenberg, Jacqueline A. Madison, Alí Duarte‐García, Jason S. Knight, Yu Zuo

**Affiliations:** ^1^ Division of Rheumatology, Department of Internal Medicine University of Michigan Ann Arbor; ^2^ ProMedica Rheumatology ProMedica Toledo Hospital Sylvania Ohio; ^3^ Department of Dermatology University of Michigan Ann Arbor; ^4^ Department of Rheumatology Mayo Clinic Rochester Minnesota; ^5^ Division of Pediatric Rheumatology, Department of Pediatrics University of Michigan Ann Arbor

## Abstract

**Objective:**

Antiphospholipid syndrome (APS) is a thromboinflammatory disorder characterized by clinical and mechanistic heterogeneity that complicates early diagnosis and hinders targeted treatment. We aimed to identify distinct molecular endotypes among antiphospholipid antibody (aPL)–positive patients using whole‐blood transcriptomics.

**Methods:**

Whole‐blood RNA sequencing was performed on 174 aPL‐positive patients, including those with primary APS (n = 102), secondary APS (n = 29), and aPL positivity without classifiable APS (n = 43). Unsupervised machine learning and immune cell deconvolution defined transcriptomic clusters and immune landscapes.

**Results:**

Four transcriptionally distinct clusters were identified. At one end of the spectrum, cluster 1 showed up‐regulation of ribosomal and metabolic pathways and down‐regulation of mechanistic target of rapamycin (mTOR), NETosis, and Hippo/interleukin‐6 (IL‐6) signaling. In contrast, cluster 4 exhibited the opposite pattern, with strong up‐regulation of mTOR, NETosis, and Hippo/IL‐6 signaling. Cluster 2 demonstrated modest enrichment in messenger RNA processing and amino acid metabolism, and cluster 3 showed biosynthetic suppression with mild Hippo/IL‐6 activation. Clinically, cluster 4 stood out with higher IgG anticardiolipin and anti–β_2_‐glycoprotein I positivity, elevated neutrophil counts, and increased urine protein‐to‐creatinine ratios. Immune deconvolution revealed distinct cell type profiles: cluster 1 was lymphoid predominant; cluster 2 had a balanced composition; cluster 3 was enriched in Treg cells, natural killer cells, macrophages, mast cells, and memory B cells; and cluster 4 was dominated by myeloid cells, including neutrophils, eosinophils, and dendritic cells. Distinct immune pathway activations were linked to clinical features, including white matter lesions, seizures, and cardiac valve disease.

**Conclusion:**

This study reveals four endotypes of aPL‐positive patients, a step toward personalized medicine for APS through pathway‐informed stratification and therapy.

## INTRODUCTION

Antiphospholipid syndrome (APS) is a thromboinflammatory, multisystem autoimmune disorder characterized by persistently positive antiphospholipid antibodies (aPL). These aPL are detected by positive testing for IgG/IgM anti–β_2_ glycoprotein I (anti‐β_2_GPI), IgG/IgM anticardiolipin (aCL), or lupus anticoagulant, the latter a functional assay that screens for various aPL.^1^ APS may occur as a standalone condition, referred to as primary APS, or it may occur alongside other systemic autoimmune diseases (typically systemic lupus erythematosus [SLE]), which is sometimes referred to as secondary APS. The clinical presentations of APS are highly diverse. Although APS was classically considered in the setting of thrombotic events and recurrent first‐trimester or late‐term pregnancy complications as defined by the 2006 APS classification criteria,^1^ additional clinical manifestations of APS were incorporated into the newest iteration of classification criteria by the American College of Rheumatology (ACR)/EULAR^2^ in 2023. These clinical manifestations include livedo reticularis/racemosa, aPL nephropathy, pulmonary hemorrhage, cardiac valve abnormalities, thrombocytopenia, and others. Furthermore, though not included in the classification criteria, patients sometimes experience other nonthrombotic clinical manifestations, such as white matter lesions, movement disorders, and cognitive dysfunction. Additionally, some individuals may have persistently positive aPL without any classifiable APS manifestations. This clinical heterogeneity presents significant challenges for both effective management and accurate risk stratification.

The pathogenesis of key aPL‐mediated clinical manifestations has been shown to involve the interplay of endothelial cells, neutrophils, monocytes, platelets, coagulation factors, and complement proteins.^3^ These findings suggest that the pathophysiology of APS is characterized by disruptions in a complex mosaic of molecular pathways, converging into overlapping clinical phenotypes.

Although the 2023 ACR/EULAR classification criteria have highlighted the clinical heterogeneity of APS, it can be challenging to achieve the precise early patient subtyping needed for proactive management and reliable outcome prediction.^2^ Leveraging modern high‐throughput “omics” technologies offers the potential to define distinct molecular endotypes—subgroups of APS driven by specific pathophysiologic mechanisms—that might inform earlier and more personalized interventions.^4^, ^5^ Advances in this area of research are essential for transforming the diagnosis, treatment, and long‐term outcomes of patients with APS.

The goal of our study was to apply integrative whole‐blood RNA transcriptomic analyses to cluster aPL‐positive patients according to their gene expression profiles. Our design focused on characterizing within‐disease heterogeneity—stratifying aPL‐positive patients—rather than defining APS‐specific transcriptional signatures relative to healthy controls. By identifying potentially pathogenic pathways, we seek to advance precision diagnosis, reveal novel therapeutic targets, and inform more personalized management strategies for APS.

## METHODS

This study complied with all relevant ethical regulations and was approved by the University of Michigan Institutional Review Board (HUM00122519, HUM00044257, and HUM00151834) and the Mayo Clinic Institutional Review Board (20‐000476).

### Human samples

A PAXgene Blood RNA tube by PreAnalytiX (REF 762165) was collected from 102 patients with primary APS, 29 patients with secondary APS, and 43 individuals with persistently positive aPL at least 12 weeks apart but without thrombotic or obstetric manifestations of APS. Our cohort was established before the publication of the 2023 ACR/EULAR criteria. As a result, some patients were classified as having APS based on multiplex aPL testing; however, all met the Sapporo criteria, with persistently positive aPL documented at least 12 weeks apart.^6^ In terms of clinical manifestations, we confirmed that all patients designated as having APS fulfilled the clinical requirements for APS classification in the 2023 criteria. However, we were not able to fully capture microvascular or nonthrombotic manifestations as outlined in the 2023 criteria.^2^ Lupus was defined according to the 2012 Systemic Lupus International Collaborating Clinics classification criteria.^7^ Calprotectin (S100A8/S100A9) levels were measured in citrated plasma using the human S100A8/S100A9 Heterodimer DuoSet enzyme‐linked immunosorbent assay (ELISA) (DY8226‐05, R&D Systems) per the manufacturer's instructions. Soluble E‐selectin and ICAM‐1 were quantified in citrated plasma by ELISA (DY724, R&D Systems).

### RNA extraction and sequencing

RNA was extracted from the samples, and quality control was performed to ensure a minimum RNA integrity number greater than 6. Whole‐blood RNA sequencing was then conducted at the University of Michigan Advanced Genomic Core using an Illumina NovaSeq 6000 sequencer. Each sample was sequenced to a depth of at least 45 million reads, generating 150 bp paired‐end reads and libraries. All RNA samples were processed, prepared for sequencing, and sequenced together in a single batch using the same library preparation protocol and sequencing run.

### RNA sequencing data analysis

RNA sequencing reads were aligned to the human genome (hg38) using STAR v2.7.11a. Gene‐level counts were obtained with quantMode GeneCounts, and all other alignment parameters were set to default. Raw counts were normalized using DESeq2 to account for sequencing depth and library size. Genes expressed in fewer than five samples were excluded as lowly expressed. A log2(x + 1) transformation was applied to the normalized counts for downstream analyses.

Principal component analysis was performed on the log‐transformed data, and the first 50 principal components were used for hierarchical clustering to generate the sample dendrogram. The choice of 50 components was guided by our prior experience and the widely accepted convention in single‐cell RNA sequencing analyses—also the default in the commonly used Seurat package—as this number reliably captures the major biologic variation while diminishing noise.^8^ Based on dendrogram structure, four distinct clusters were identified. Differential expression analysis among these clusters was conducted using DESeq2 with default parameters. Genes with adjusted *P* values <0.05 were considered significantly differentially expressed and were subjected to enrichment analysis using Metascape.

To identify coexpression patterns and their associations with sample traits, weighted gene coexpression network analysis (WGCNA) was performed on the log‐transformed data. A signed weighted correlation network was constructed using pairwise Pearson correlations, and an adjacency matrix was generated using a soft‐thresholding power of 10. This matrix was transformed into a topological overlap matrix (TOM) to quantify network connectivity. Genes were hierarchically clustered based on TOM dissimilarity, and gene modules were identified using dynamic tree cutting with a minimum module size of 50. Similar modules were merged, resulting in 22 distinct modules. Module eigengenes were correlated with clinical traits using Pearson correlation, and functional enrichment of each module was assessed via Metascape.

Pathway enrichment was performed using Metascape (www.metascape.org). For each WGCNA module, the corresponding gene list was uploaded and analyzed with default Metascape settings, which simultaneously query multiple annotation databases, including Gene Ontology (GO) Biological Process, GO Cellular Component, GO Molecular Function, KEGG Pathways, Reactome Gene Sets, and CORUM protein complexes. Metascape applies a false discovery rate correction and defines statistical significance as enrichment q < 0.05. Only pathways meeting this built‐in false discovery rate threshold were considered significant and are reported. Because some modules share genes and can therefore produce overlapping enriched pathways, Metascape reduces redundancy by clustering enriched terms into nonoverlapping groups based on gene overlap. Enrichment analysis was performed independently for each module, and our interpretation emphasizes robust, recurrent pathways that appeared across multiple modules and remained significant after adjusting for false discovery rates.

Cell type composition was estimated with CIBERSORTx using the LM22 signature matrix to deconvolute immune cell populations in each sample. LM22 reference matrix was used because it is a widely validated immune cell deconvolution panel optimized for peripheral blood transcriptomes.^9^, ^10^ Because this was an exploratory study aimed at identifying molecular and cellular heterogeneity in APS, we did not perform a priori power calculations for immune cell deconvolution. To minimize the potential impact of the smaller cluster size, which could reduce sensitivity for detecting subtle shifts in cell proportions, we focused our interpretation on moderate to large changes (≥15%–20%) in immune cell fractions. Pearson correlations were then computed between pathway enrichment scores and cell type abundance estimates. We applied multiple‐testing corrections throughout the study using the Benjamini–Hochberg false discovery rate method for differential expression, WGCNA module–trait correlations, immune cell deconvolution correlations, and the built‐in false discovery rate adjustment in Metascape for pathway enrichment analyses.

### Pathway‐specific gene enrichment score calculation

Gene sets for neutrophil extracellular traps (NETs) and Hippo pathways were assembled from published studies, including our own autoimmune transcriptomic work, as well as curated pathway databases and public gene libraries.^11^ From these sources, we retained only genes that were reliably detected in our peripheral blood RNA sequencing data set and consistently represented in prior blood‐based signatures. For each sample, the pathway gene score was then computed as the mean normalized expression of all genes in the set, yielding a single quantitative measure of relative pathway activity.

Together, these integrative analyses aimed to reveal key transcriptomic signatures and potential biomarkers across primary APS, secondary APS, and persistently aPL‐positive individuals without classifiable APS.

### Data availability

Transcriptomic data are not publicly available due to privacy and consent restrictions. All other data supporting the findings of this study, including the main text and supplementary materials, are available from the corresponding author upon reasonable request (Yu Zuo, yzu@umich.edu).

## RESULTS

### Molecularly stratifying aPL‐positive patients reveals distinct immune signaling pathways

**Figure 1:**
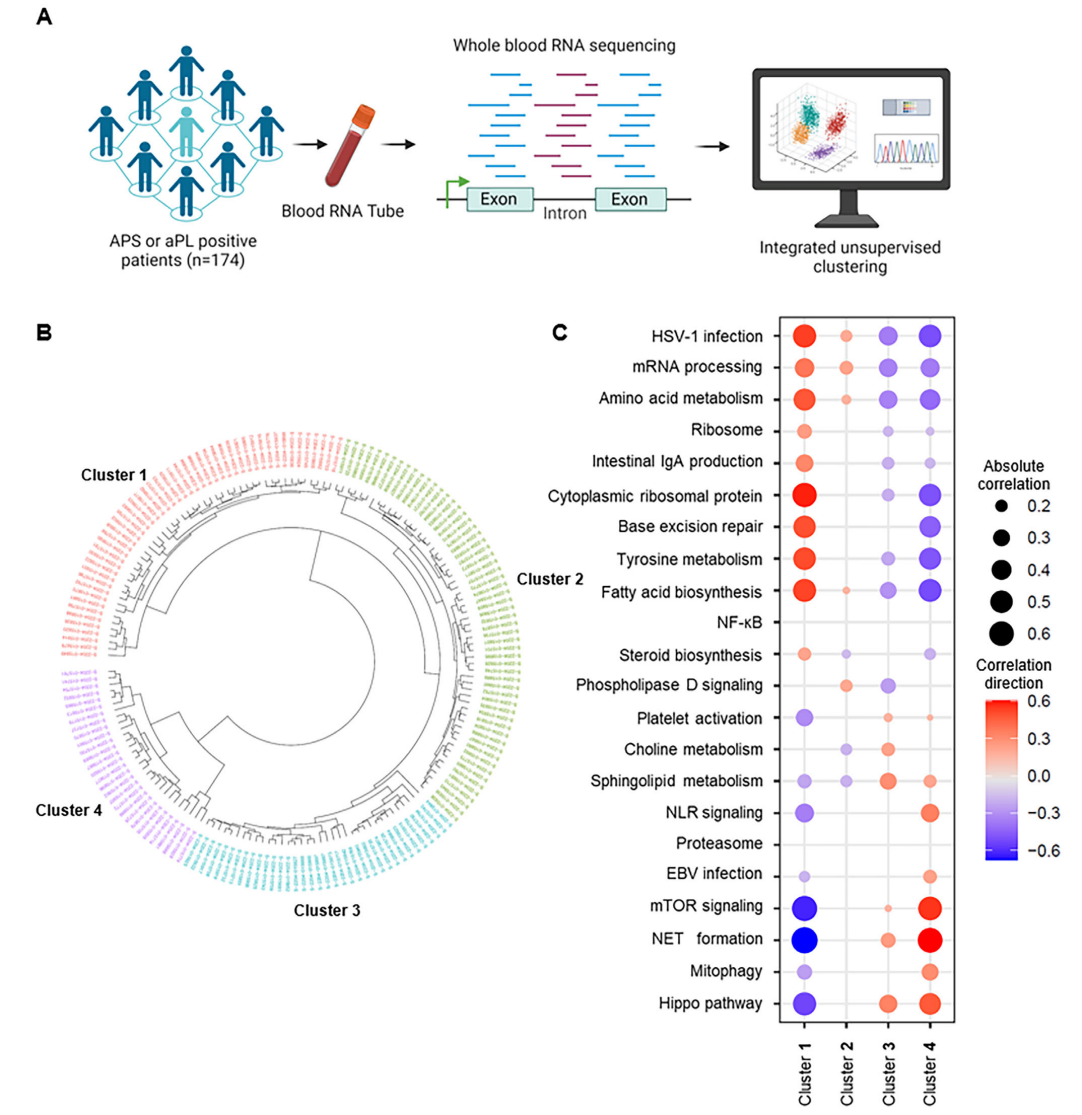
Molecularly stratifying aPL‐positive patients. (A) Schematic illustration of study design and whole‐blood RNA sequencing. The figure was created at www.biorender.com. (B) Unsupervised hierarchical clustering stratified aPL‐positive patients into four distinct clusters, each represented by a different color. Each code within the clusters corresponds to an individual patient's RNA sample. (C) Dot plots illustrate the associations between pathway‐specific gene modules and each cluster of aPL‐positive patients. Only statistically significant associations are displayed. The specific genes composing each pathway‐associated module are detailed in the Supplemental Data. aPL, antiphospholipid antibody; APS, antiphospholipid syndrome; EBV, Epstein‐Barr virus; HSV, herpes simplex virus; mRNA, messenger RNA; mTOR, mammalian target of rapamycin; NET, neutrophil extracellular trap; NLR, nucleotide‐binding oligomerization domain–like receptor.

Whole‐blood RNA sequencing was performed on 174 patients, including 102 with primary APS, 29 with secondary APS (all meeting SLE classification criteria^7^), and 43 with persistently positive aPL but without classifiable APS manifestations. Log‐transformed whole‐blood gene expression data from these patients were subjected to principal component analysis, and the first 50 principal components were used to perform hierarchical clustering. A sample dendrogram was constructed based on this analysis, from which four distinct patient clusters were identified (Figure 1A and B). To validate this four‐cluster solution, we performed consensus clustering, which likewise supported the four‐cluster structure and demonstrated stable cluster assignments. To further explore transcriptomic differences, we used WGCNA, which identified 22 distinct gene modules. These modules were subsequently analyzed to determine their enrichment in specific biologic pathways and processes, providing insight into the molecular landscape underlying each cluster (Figure 1C). Relative to the other aPL‐positive patient clusters, cluster 1 showed the strongest up‐regulation of pathways involved in cytoplasmic ribosomal proteins, base excision repair, tyrosine metabolism, and fatty acid biosynthesis. Conversely, it was down‐regulated in mammalian target of rapamycin (mTOR) signaling, NET formation, and Hippo/interleukin‐6 (IL‐6) signaling pathways. Cluster 2 demonstrated modest relative up‐regulation of messenger RNA (mRNA) processing and amino acid–metabolism pathways. Cluster 3 exhibited modest relative down‐regulation of mRNA processing, amino acid biosynthesis, and fatty acid biosynthesis, accompanied by mild relative up‐regulation of the Hippo/IL‐6 signaling pathway. Cluster 4 displayed the inverse pattern of cluster 1, with strong relative up‐regulation of mTOR signaling, NET formation, and Hippo/IL‐6 signaling and relative down‐regulation of cytoplasmic ribosomal proteins, base excision repair, tyrosine metabolism, and fatty acid biosynthesis.

We performed sensitivity analyses to ensure our findings were not driven by confounding factors. First, we included immune cell–proportion estimates from CIBERSORTx (LM22) as covariates in the linear models testing the association between each WGCNA module and cluster membership; this adjustment, after correcting for multiple comparisons, did not alter the results, as the NET formation and Hippo/IL‐6 signaling gene modules remained highly and significantly associated with cluster 4. To evaluate the influence of APS subtypes (primary APS, secondary APS, or aPL‐only without classifiable APS), we added subtype as a covariate and again found both modules were strongly associated with cluster 4; cluster membership also remained unchanged when the analysis was restricted to patients with primary APS. Finally, to test for possible medication effects, we adjusted for hydroxychloroquine (HCQ) use and for a combined indicator of other immunosuppressive therapy (mycophenolate, rituximab, methotrexate, azathioprine, leflunomide, or belimumab). Both the NET formation and Hippo/IL‐6 signaling gene modules remained highly significantly associated with cluster 4 after these adjustments (NET formation: adjusted cluster *P* = 3.86 × 10^−41^; Hippo/IL‐6 signaling: adjusted cluster *P* = 4.98 × 10^−24^), and the results were unchanged even after excluding all patients receiving HCQ or any immunosuppressive therapy. These analyses demonstrate that the strong association of these gene modules with cluster 4 is robust and independent of immune cell composition, APS subtype, and medication use.

Table 1 summarizes the clinical and demographic features of these patients across four transcriptomic clusters. Although the clusters showed similar distributions of age and sex, as well as history of thrombosis, obstetric morbidity, thrombocytopenia, livedo reticularis, cardiac valve disease, and APS nephropathy, notable differences still emerged. Cluster 4 had a higher prevalence of IgG aCL and IgG anti‐β_2_GPI positivity compared to clusters 1 to 3. Individuals in cluster 4 also had elevated absolute neutrophil counts, circulating calprotectin levels—a marker of neutrophil activation and NET formation—and urine protein‐to‐creatinine ratios. Patients in cluster 4 were also more likely to receive immunosuppressive therapy, whereas circulating E‐selectin levels, a marker of endothelial activation, did not differ significantly among clusters.

**Table 1:** Clinical and demographic characteristics of aPL‐positive patients across four clusters (N = 174)*

	Cluster 1 (n = 50)	Cluster 2 (n = 59)	Cluster 3 (n = 36)	Cluster 4 (n = 29)	*P* value
Diagnoses, n (%)					
Primary APS (thrombotic or obstetric manifestations)	30 (60.0)	34 (57.6)	20 (55.6)	18 (62.1)	0.976
aPL positive without classifiable APS	12 (24.0)	10 (17.0)	12 (33.3)	9 (31.0)	0.233
Demographics					
Age, mean ± SD, y	43 ± 13	46 ± 18	46 ± 14	45 ± 17	0.879
Male, n (%)	15 (30.0)	15 (25.4)	12 (33.3)	9 (31.0)	0.859
aPL positivity profile, n (%)					
IgG aCL	26 (52.0)	40 (67.8)	26 (89.7)	22 (75.7)	**0.022**
IgM aCL	27 (54.0)	26 (44.1)	12 (33.3)	11 (37.9)	0.076
IgG anti‐β_2_GPI	29 (58.0)	42 (71)	26 (72)	26 (90)	**0.005**
IgM anti‐β_2_GPI	26 (52.0)	26 (44.1)	12 (33.3)	14 (48.3)	0.405
Lupus anticoagulant	20 (40.0)	34 (57.6)	21 (58.3)	16 (55.2)	0.149
Triple positive^a^	12 (24.0)	26 (44.1)	15 (41.7)	12 (41.4)	0.116
Laboratory studies, mean ± SD					
Neutrophil count, 10^3^/μL^b^	3.29 ± 1.89	4.48 ± 1.91	4.28 ± 0.35	6.29 ± 2.16	**<0.0001**
CRP, mg/dL	0.32 ± 0.36	0.52 ± 0.54	0.80 ± 0.72	0.69 ± 0.81	0.050
C3, mg/dL	74.35 ± 17.62	77.79 ± 17.08	78.76 ± 15.82	76.76 ± 21.28	0.770
C4, mg/dL	19.85 ± 4.52	20.38 ± 5.07	20.21 ± 4.54	19.39 ± 6.02	0.871
Platelet count, 10^3^/μL^b^	241.11 ± 78.25	263.35 ± 90.84	221.68 ± 77.97	216.21 ± 73.85	0.074
Creatinine, mg/dL^b^	1.05 ± 0.65	0.98 ± 0.36	1.03 ± 0.33	1.00 ± 0.47	0.679
Urine protein/creatinine ratio^b^	0.09 ± 0.14	0.41 ± 0.91	0.31 ± 0.52	0.49 ± 0.90	**0.043**
E‐selectin, pg/mL	29,530 ± 15,851.60	32,143.89 ± 21,024.42	34,875.52 ± 17,416.36	38,409.11 ± 21,422.65	0.284
Calprotectin, μg/mL	1.28 ± 1.17	1.74 ± 1.54	2.45 ± 1.65	3.15 ± 3.23	**<0.001**
Clinical history, n (%)					
Venous thrombosis	22 (44)	29 (49.2)	22 (61.1)	14 (48.3)	0.386
Arterial thrombosis	10 (20.0)	15 (25.4)	6 (16.7)	5 (17.2)	0.580
Pregnancy morbidity	12 (24.0)	7 (11.9)	5 (13.9)	4 (13.8)	0.234
Cardiac valve disease	5 (10.0)	7 (11.9)	8 (22.2)	6 (20.7)	0.091
Livedo reticularis	18 (36.0)	13 (22.0)	9 (25.0)	12 (41.4)	0.77
APS nephropathy	4 (8.0)	4 (6.8)	2(5.6)	1 (3.4)	0.411
Immunosuppressive medications, n (%)					
Hydroxychloroquine	26 (52.0)	30 (50.8)	20 (55.6)	20 (69.0)	**0.03**
Other immunosuppressive medications^c^	7 (14)	11 (18.6)	8 (22)	9 (31)	**0.03**
NET and Hippo scores, mean ± SD					
NET score	−2.23 ± 0.96	−0.46 ± 1.89	0.47 ± 2.36	4.21 ± 6.67	**<0.0001**
Hippo score	−5.04 ± 3.72	−0.29 ± 4.86	0.15 ± 5.49	9.1 ± 9.52	**<0.0001**

* Intergroup differences for categorical variables were assessed using the Jonckheere–Terpstra trend test, whereas differences for continuous variables were evaluated using one‐way analysis of variance for normally distributed data and the Kruskal–Wallis test for skewed distributions. Bold *P* values indicate *p* < 0.05. aCL, anticardiolipin; anti‐β_2_GPI, anti–β_2_ glycoprotein I; aPL, antiphospholipid antibody; APS, antiphospholipid syndrome; CRP, C‐reactive protein; NET, neutrophil extracellular trap.

^a^ Triple positive includes positive lupus anticoagulant, positive IgG or IgM aCL, and IgG or IgM positive anti‐β_2_GPI.

^b^ Platelet counts, absolute neutrophil counts, and serum creatinine levels were obtained from clinical laboratory test results collected on the same day as the research RNA samples. Among the 174 participants, 123 had these clinical laboratory tests performed on the corresponding date. Similarly, urine protein‐to‐creatinine ratios were extracted from clinical laboratory data when available on the same day as RNA sample collection; 124 of 174 participants had this measurement.

^c^ Other immunosuppressive medications: mycophenolate, rituximab, methotrexate, azathioprine, leflunomide, or belimumab.

Across the 22 gene modules, NET formation and Hippo/IL‐6 signaling pathways showed some of the most pronounced positive and negative correlations (Figure 1C). To explore this further, we generated pathway‐specific enrichment scores by selecting genes ranked by their contribution to NETs pathway enrichment (5 genes) and Hippo pathway activation (11 genes), based on our prior work and public gene libraries (Supplemental Table 1). These NET and Hippo enrichment scores were applied across the patient cohort. We found that both NET and Hippo scores varied significantly among the clusters. Cluster 4 had the highest enrichment scores for both pathways, whereas cluster 1 had the lowest (Table 1). A higher NET score was associated with increased levels of circulating calprotectin (a marker of neutrophil activation and NET formation), elevated absolute neutrophil counts, higher positivity for IgG aCL and IgG anti‐β_2_GPI, and increased urine protein‐to‐creatinine ratios (Supplemental Table 2). Similarly, a higher Hippo score correlated with elevated levels of calprotectin, C‐reactive protein (CRP), E‐selectin (a marker of endothelial activation), absolute neutrophil counts, and urine protein‐to‐creatinine ratios (Supplemental Table 2).

### Immune cell composition across the four clusters

**Figure 2:**
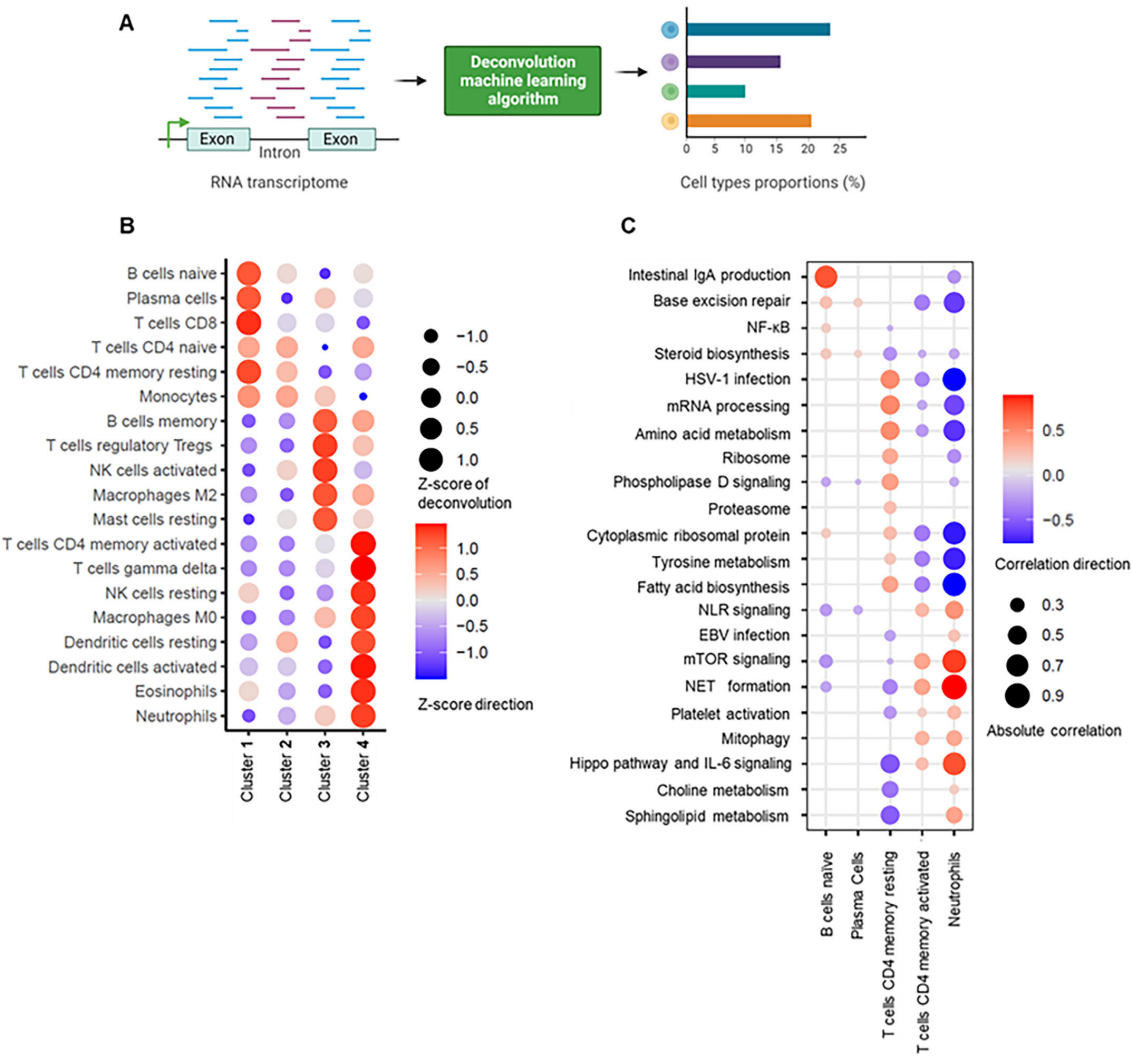
Deconvolution analysis unveils the immune cell composition across four distinct clusters. (A) Schematic illustration of deconvolution analysis of whole‐blood transcriptomic data. Cell type deconvolution was performed using CIBERSORTx with the LM22 (22 immune cell types) as the signature matrix. The figure was created at www.biorender.com. (B) Dot plot demonstrating associations between immune cell types and each cluster of aPL‐positive patients. (C) Dot plot showing associations between pathway‐specific gene modules and immune cell types. Only associations that remain statistically significant after multiple‐comparison adjustment are displayed. aPL, antiphospholipid antibody; APS, antiphospholipid syndrome; EBV, Epstein‐Barr virus; HSV, herpes simplex virus; IL, interleukin; mRNA, messenger RNA; mTOR, mammalian target of rapamycin; NET, neutrophil extracellular trap; NK, natural killer; NLR, nucleotide‐binding oligomerization domain–like receptor.

Deconvolution analysis revealed distinct immune cell type distributions among the four clusters (Figure 2A and B). Cluster 1 was enriched in lymphoid cells, including B cells, plasma cells, CD4^+^ T cells, and CD8^+^ T cells. Cluster 2 displayed a relatively uniform distribution of all analyzed immune cell types. Cluster 3 showed enrichment in Treg cells, natural killer (NK) cells, macrophages, mast cells, and memory B cells. Cluster 4 was characterized by a predominance of myeloid cells, including neutrophils, eosinophils, dendritic cells, macrophages, NK cells, and T cells. We also observed several significant associations between specific immune cell types and pathway‐specific gene modules (Figure 2C). Neutrophils demonstrated strong positive correlations with NET formation, Hippo/IL‐6 signaling, and mTOR signaling pathways and significant negative correlations with fatty acid biosynthesis, tyrosine metabolism, and cytoplasmic ribosomal protein pathways. B cells were positively correlated with intestinal IgA production, whereas plasma cells did not show notable associations with any gene modules. Resting T cells exhibited moderate negative correlations with Hippo/IL‐6 signaling, sphingolipid metabolism, and choline metabolism, alongside moderate positive correlations with mRNA processing and amino acid metabolism (Figure 2C). To validate our deconvolution estimates, we correlated CIBERSORTx‐derived neutrophil proportions with absolute neutrophil counts obtained from clinical complete blood cell counts. The correlation was moderate to strong and highly significant, providing confirmation of the accuracy of our deconvolution results (Supplemental Figure 1).

### The association of gene modules with nonthrombotic/nonobstetric clinical manifestations among patients with APS and aPL‐positive patients

Analysis of the associations between pathway‐specific gene modules and nonthrombotic, nonobstetric clinical manifestations of APS revealed several statistically significant findings (Supplemental Figure 2). White matter lesions were linked to up‐regulation of genes involved in NF‐κB signaling and cytoplasmic ribosomal proteins. Seizures were associated with increased expression of genes related to choline and sphingolipid metabolism. Cardiac valve disease showed up‐regulation of genes associated with Hippo/IL‐6 signaling and NET formation, alongside down‐regulation of genes involved in intestinal IgA production (Supplemental Figure 2).

## DISCUSSION

Using whole‐blood RNA sequencing integrated with unsupervised machine learning and immune cell deconvolution, our study stratified patients with APS or persistently positive aPL across diverse clinical presentations into four biologically distinct clusters. These clusters were defined by coordinated gene expression modules and predicted immune cell compositions. Cluster 1 was marked by up‐regulation of cytoplasmic ribosomal proteins, base excision repair, tyrosine metabolism, and fatty acid biosynthesis pathways, with down‐regulation of mTOR signaling, NET formation, and Hippo/IL‐6 signaling. In contrast, cluster 4 displayed the inverse pattern, with strong up‐regulation of mTOR signaling, NET formation, and Hippo/IL‐6 pathways. Cluster 2 showed modest enrichment in mRNA processing and amino acid–metabolism pathways, whereas cluster 3 exhibited relative suppression of similar biosynthetic pathways, alongside mild up‐regulation of Hippo/IL‐6 signaling. Cluster 4 also stood out for its clinical associations—higher rates of IgG aCL and anti‐β_2_GPI positivity, elevated neutrophil counts, and increased urine protein‐to‐creatinine ratios. Immune deconvolution further revealed distinct cellular landscapes: cluster 1 had a lymphoid‐predominant profile; cluster 2 displayed a balanced mix; cluster 3 was enriched in Treg cells, NK cells, macrophages, mast cells, and memory B cells; and cluster 4 was myeloid‐skewed, with neutrophils, eosinophils, and dendritic cells. Our cohort of 174 aPL‐positive patients represents, to our knowledge, the largest whole‐blood RNA sequencing study in APS to date. Although cluster 4 included only 29 patients, several analyses confirm adequate power. Associations of cluster 4 with the NET formation and Hippo/IL‐6 signaling modules were exceptionally strong (*P* < 1 × 10^−40^) and remained highly significant after 5,000 permutation tests (empirical *P* < 1 × 10^−3^). These findings were robust to sensitivity analyses; removing 20% of samples preserved significance in >95% of resamples, and even downsampling cluster 4 to 10 patients retained significance in 100% of runs. A formal one‐way analysis of variance power analysis further indicated >0.99 power to detect the observed effect sizes. Thus, despite its smaller size, cluster 4 is sufficiently powered to support the stability of these associations.

Whole‐blood RNA sequencing has previously been applied in APS research to compare transcriptomic profiles between patients and healthy controls, consistently revealing marked gene expression changes—particularly in interferon‐regulated genes (IRGs).^12^, ^13^, ^14^, ^15^, ^16^ For instance, a study of 62 patients with thrombotic primary APS and 29 healthy controls identified 34 differentially expressed genes, 33 of which were up‐regulated by at least two‐fold, including 14 IRGs—highlighting the prominent role of interferon signaling in APS pathogenesis.^13^ Gene expression profiling in that study distinguished patients with APS from controls with 79% accuracy, which improved to 82% when analysis was focused exclusively on IRGs, underscoring their promise as potential biomarkers. In contrast, our study did not detect significant variation in IRGs signatures within the APS cohort. This is likely due to differences in comparison strategies. Although patients with APS typically show elevated baseline interferon activity relative to healthy individuals, the variation within the APS or aPL‐positive group is likely more limited, making it difficult to detect statistically significant differences across subgroups. Building on prior work, our study uniquely applies whole‐blood RNA sequencing in combination with unsupervised clustering to stratify aPL‐positive individuals into four transcriptionally distinct subgroups. This approach moves beyond conventional primary versus secondary APS classification and reveals underlying molecular heterogeneity. The presence of such distinct molecular signatures supports the concept that APS comprises a spectrum of immune and vascular phenotypes. This stratification highlights the need for more personalized approaches to APS care, guided by the dominant immunopathogenic mechanisms in each subgroup.

Our study reinforces the role of neutrophil‐driven inflammation in a subset of APS patients, further implicating NETs in disease pathogenesis. Pathway enrichment analysis revealed significant up‐regulation of NET formation genes in cluster 4, whereas these pathways were down‐regulated in cluster 1. NETs—extracellular DNA and chromatin structures released by activated neutrophils—have been implicated in promoting vascular inflammation, endothelial damage, and hypercoagulability. Prior studies, including our own, have demonstrated that aPL directly trigger NETosis and that increased circulating NET‐associated biomarkers are associated with thrombotic risk in APS.^3^, ^17^ Transcriptomic analysis of APS neutrophils further supports this link, revealing an activated neutrophil signature that enhances adhesion and NET formation.^18^ Our study builds on these findings by showing that patients with APS in cluster 4 have elevated NET enrichment scores, which correlate with increased circulating calprotectin levels—a marker of neutrophil activation and NET formation—as well as a higher protein‐to‐creatinine ratio, indicating renal damage and potential inflammation. This aligns with a recent transcriptomic study of APS kidneys, which revealed up‐regulated NET‐related genes in affected kidneys.^19^ Additionally, patients in cluster 4 had higher levels of IgG aCL and IgG anti‐β_2_GPI. Notably, this mirrors a recent lupus transcriptomic study showing that aPL positivity in patients with lupus is associated with up‐regulation of neutrophil and myeloid gene modules,^20^ further supporting the hypothesis that neutrophil‐driven inflammation exacerbates APS autoimmunity.

Beyond NETosis, the up‐regulation of Hippo/IL‐6 signaling in cluster 4 offers potential insights into APS‐associated immune remodeling, particularly in the context of myeloid cell activation and inflammation. The Hippo pathway regulates the transcriptional coactivators YAP and TAZ, which, when activated, translocate to the nucleus and drive proinflammatory gene expression programs, including IL‐6 production, and enhance immune cell survival and chemotaxis.^21^, ^22^ In myeloid cells such as neutrophils and macrophages, YAP/TAZ activity has been shown to promote inflammatory polarization, prolong cell lifespan, and support tissue infiltration—all features consistent with the innate immune activation observed in APS.^21^, ^22^ In our study, elevated expression of Hippo pathway genes in cluster 4 was accompanied by increased calprotectin and CRP levels, supporting a potential mechanistic link between Hippo signaling and systemic inflammation. Additionally, Hippo signaling is known to be activated by mechanical and cellular stress, which is particularly relevant in APS, in which endothelial dysfunction is a key feature of vascular pathology. We observed a positive association between Hippo pathway enrichment and circulating E‐selectin levels—a marker of endothelial activation—suggesting that this signaling axis may represent an immune cell response to vascular stress. Taken together, these findings suggest that Hippo pathway activation may contribute to a proinflammatory, myeloid‐driven endotype in a subset of patients with APS. This pathway could serve as a potential new therapeutic target to mitigate thromboinflammatory risk, and future studies should investigate its functional role in APS pathogenesis more directly.

Our study further highlights the relationship between immune cell composition and the molecular heterogeneity of APS. Deconvolution analysis of immune cells highlighted variation in immune system involvement across APS subtypes. Patients in cluster 4 exhibited a dominant myeloid signature, characterized by increased neutrophils, eosinophils, and dendritic cells, whereas cluster 1 was enriched in lymphoid cells, including B cells, plasma cells, and CD8^+^ T cells. These findings reinforce the notion that APS pathogenesis is associated with distinct immune mechanisms in different patient subsets. Although prior research has focused on B cell–mediated autoantibody production in APS, our data emphasize the critical role of innate immune cells in the disease process. This myeloid‐driven inflammatory signature further supports the involvement of neutrophils in APS‐related vascular complications and thrombosis, suggesting potential avenues for targeted therapeutic intervention.

Our findings suggest that nonthrombotic APS manifestations are associated with distinct gene expression profiles, providing mechanistic insights that align with prior research. Seizures were linked to up‐regulation of genes involved in choline and sphingolipid metabolism—pathways previously implicated in seizure generation, epileptogenesis, and drug‐resistant epilepsy across human studies, animal models, and monogenic metabolic disorders.^23^, ^24^, ^25^ Cardiac valve disease was associated with increased expression of genes related to Hippo and IL‐6 signaling, consistent with growing evidence that persistent IL‐6 elevation plays a direct pathogenic role in vascular and degenerative valve disease rather than serving solely as a marker of inflammation.^26^, ^27^ Together, these findings support the biologic relevance of these pathways in APS‐related organ damage.

We acknowledge several limitations in our study. Chief among them is the absence of healthy control samples. Because our study was specifically designed to stratify aPL‐positive patients and examine within‐disease heterogeneity, the study does not directly define APS‐specific transcriptional signatures relative to healthy individuals. Next, relying on whole‐blood transcriptomics may mask cell‐specific contributions to APS pathogenesis. Single‐cell RNA sequencing could provide a more precise view of immune cell involvement. Additionally, our cross‐sectional design limits the ability to assess dynamic gene expression changes. Longitudinal studies tracking transcriptomic shifts over time and responses to treatment could offer deeper insights into disease progression. We also acknowledge that estimating cell type composition with CIBERSORTx may introduce indirect compositional effects, so correlations between gene modules and cell types should be interpreted with caution. The underlying biologic relationships will require validation using independent experimental approaches. The clinical significance of the four molecular endotypes requires further validation. To gain deeper insight into APS pathogenesis, future studies should investigate the direct contribution of the novel gene expression modules identified in our study. Pathways such as Hippo signaling that have not been previously implicated in APS warrant further investigation to clarify their role in driving the underlying immunologic and vascular abnormalities characteristic of the disease. Finally, the derived NETs and Hippo pathway scores were used solely in an exploratory framework to illustrate relative pathway activity across APS/aPL‐positive patient clusters. They are not intended as validated diagnostic tools or definitive pathway signatures. Although these gene sets capture key elements of NETosis and Hippo signaling, they are not comprehensive. Future studies incorporating broader gene panels and orthogonal validation methods—such as protein‐level measurements or functional NET assays—will be necessary to confirm these findings and assess their potential clinical utility.

In summary, our study establishes the potential to stratify patients with APS and aPL‐positive patients based on whole‐blood transcriptomic profiles, uncovering biologically distinct subgroups with diverse immune activation patterns and clinical features. This level of stratification could not have been achieved through clinical or serologic data alone and offers clinicians a previously inaccessible layer of insight into APS heterogeneity. Notably, the identification of NETosis and Hippo pathway activation as key differentiators among patient subgroups points to possible novel therapeutic targets and could lay the groundwork for more personalized approaches to APS management. Looking ahead, integration of single‐cell RNA sequencing and proteomic data will be critical to refine these molecular signatures and to delineate the specific cellular drivers of APS immunopathogenesis. Furthermore, longitudinal studies are needed to assess the temporal stability of these transcriptomic clusters and their relationship to disease progression and treatment response. By leveraging transcriptomic profiling to define biologically meaningful APS endotypes, we hope to move the field closer to precision medicine strategies tailored to the underlying immune mechanisms of individual patients.

## AUTHOR CONTRIBUTIONS

All authors contributed to at least one of the following manuscript preparation roles: conceptualization AND/OR methodology, software, investigation, formal analysis, data curation, visualization, and validation AND drafting or reviewing/editing the final draft. As corresponding author, Dr Zuo confirms that all authors have provided the final approval of the version to be published and takes responsibility for the affirmations regarding article submission (eg, not under consideration by another journal), the integrity of the data presented, and the statements regarding compliance with institutional review board/Declaration of Helsinki requirements.

## Supporting information


**Disclosure Form**:

**Data S1** Supporting Information

**Data S2** Supporting Information
