# Supplementary material for: Molecular Stratification of Antiphospholipid Syndrome Through Integrative Analysis of the Whole‐Blood RNA Transcriptome
Source: Arthritis Rheumatol. 2026 Feb 13;78(5):1124–33. doi: 10.1002/art.70021 (PMC13129634; doi:10.1002/art.70021)
Supplement: Supplementary file 2 — Data S1 Supporting Information [file ART-78-1124-s002.docx]

Supplemental Materials

**Molecular stratification of antiphospholipid syndrome patients through integrative analysis of the whole-blood RNA transcriptome**

Ambati et al.

| **Supplemental Table 1: Genes selected for NETs formation and Hippo pathway score** | |
| --- | --- |
| **NETs score genes** | **Hippo score genes** |
| AZU1 | DLGAP1-AS2 |
| CR1 | MOB1A |
| CTSG | PIK3CB |
| ELANE | PIWIL4 |
| FCGR3A | PSTPIP2 |
|  | SERPINB1 |
|  | SLPI |
|  | SMAD4 |
|  | SPINK7 |
|  | SPINK8 |
|  | TPI1P2 |

| **Supplemental Table 2: Correlation between various clinical parameters and NETs formation score and Hippo pathway score (p values are not adjusted for multiple comparisons)** | | | | |
| --- | --- | --- | --- | --- |
|  | **NETs score** | | **Hippo score** | |
|  | Spearman’s correlation | | Spearman’s correlation | |
| **Variables** | **r** | **p** | **r** | **p** |
| Calprotectin | 0.3261 | **<0.0001** | 0.3055 | **0.0001** |
| C-reactive protein | 0.09215 | 0.38 | 0.2164 | **0.038** |
| E-selectin | 0.06042 | 0.46) | 0.2826 | **0.0004** |
| Absolute neutrophil count | 0.4117 | **<0.0001** | 0.4265 | **<0.0001** |
| aCL IgG | 0.2553 | **0.0019** | 0.1440 | 0.084 |
| aCL IgM | 0.02901 | 0.73 | -0.01292 | 0.88 |
| aB2GPI IgG | 0.2239 | **0.0053** | 0.03214 | 0.69 |
| aB2GPI IgM | -0.005367 | 0.95 | 0.01767 | 0.83 |
| Urine protein/creatinine ratio | 0.2212 | **0.042** | 0.2197 | **0.043** |
| aCL=anticardiolipin, aβ2GPI=anti-beta-2 glycoprotein I | | | | |


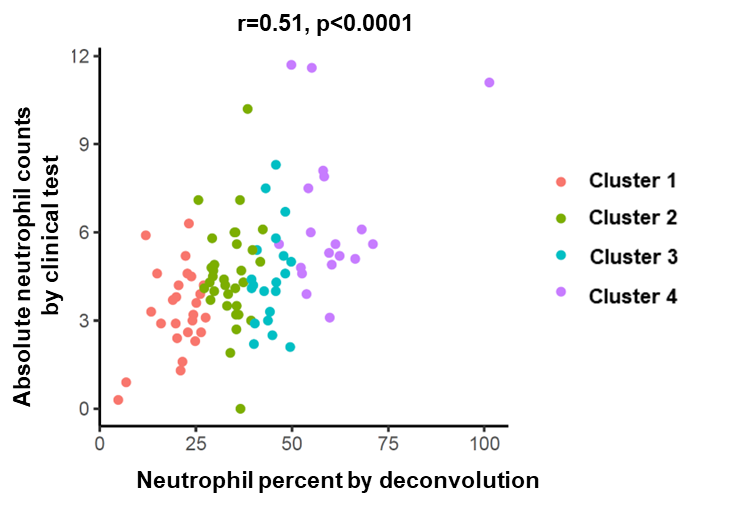


**Supplemental Figure 1.** **Correlation between absolute neutrophil counts and neutrophil proportions estimated by CIBERSORTx deconvolution**. Assessed by Pearson’s correlation.


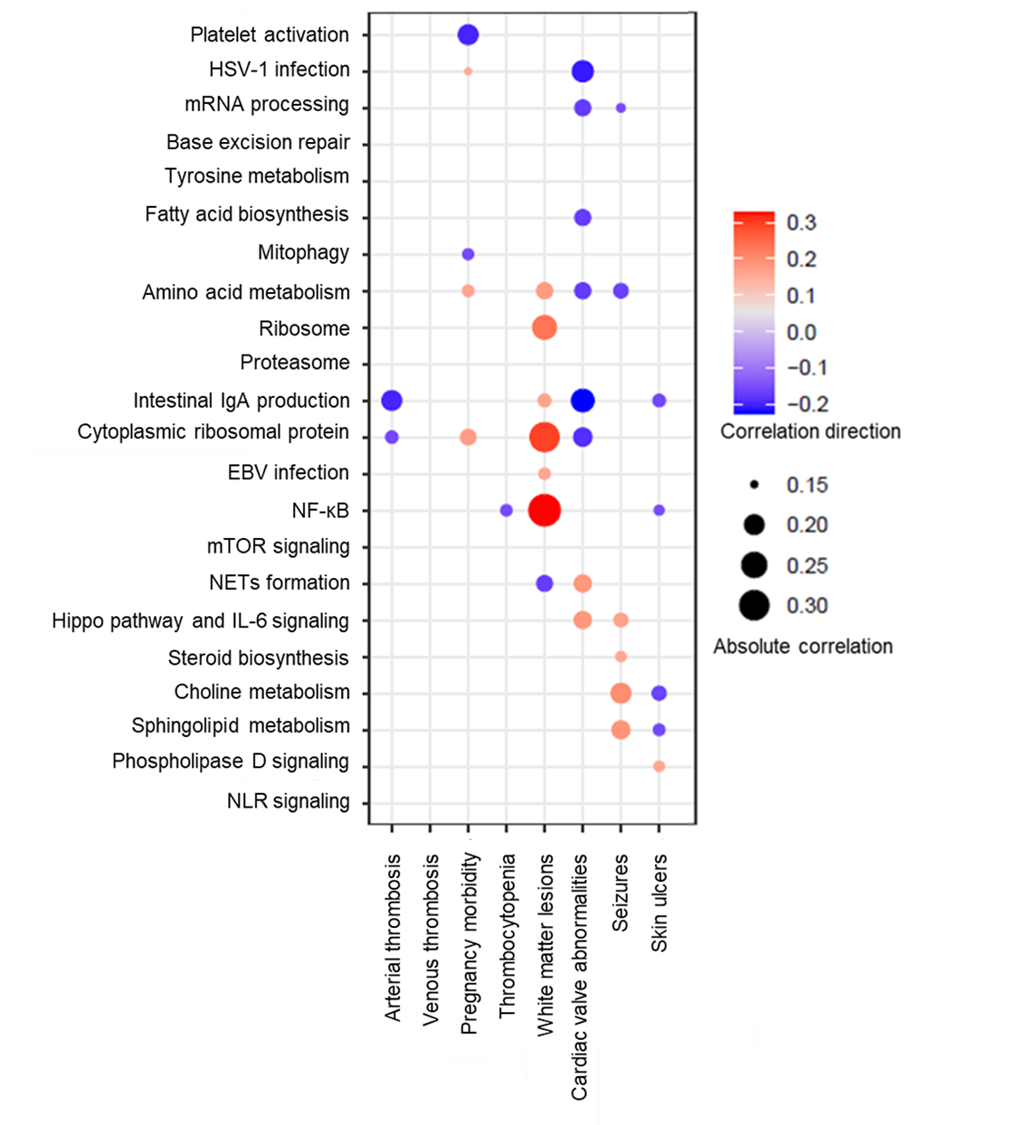


**Supplemental Figure 2.** **Gene modules’ correlation with clinical manifestations**. Dot plot demonstrating associations between pathway-focused gene expression modules and clinical manifestations of APS (both “criteria” and “non-criteria” manifestations).
